# Transposable Elements: Epigenetic Silencing Mechanisms or Modulating Tools for Vertebrate Adaptations? Two Sides of the Same Coin

**DOI:** 10.3390/ijms241411591

**Published:** 2023-07-18

**Authors:** Elisa Carotti, Federica Carducci, Marco Barucca, Adriana Canapa, Maria Assunta Biscotti

**Affiliations:** Dipartimento di Scienze della Vita e dell’Ambiente, Università Politecnica delle Marche, 60131 Ancona, Italy; e.carotti@pm.univpm.it (E.C.); m.barucca@univpm.it (M.B.); a.canapa@univpm.it (A.C.); m.a.biscotti@univpm.it (M.A.B.)

**Keywords:** transposable elements, vertebrates, evolution, silencing mechanisms, adaptation

## Abstract

Transposable elements constitute one of the main components of eukaryotic genomes. In vertebrates, they differ in content, typology, and family diversity and played a crucial role in the evolution of this taxon. However, due to their transposition ability, TEs can be responsible for genome instability, and thus silencing mechanisms were evolved to allow the coexistence between TEs and eukaryotic host-coding genes. Several papers are highlighting in TEs the presence of regulatory elements involved in regulating nearby genes in a tissue-specific fashion. This suggests that TEs are not sequences merely to silence; rather, they can be domesticated for the regulation of host-coding gene expression, permitting species adaptation and resilience as well as ensuring human health. This review presents the main silencing mechanisms acting in vertebrates and the importance of exploiting these mechanisms for TE control to rewire gene expression networks, challenging the general view of TEs as threatening elements.

## 1. Introduction

Barbara McClintock used *Zea mays* to provide evidence that genes were physically located on chromosomes when studying genetic recombination [1]. In particular, she described the crossing-over phenomenon, consisting of the exchange of genetic material between homologous chromosomes during meiosis. These studies allowed her to discover the existence of transposable elements (TEs), i.e., portions of DNA capable of moving from one chromosome to another (see Section 2). This evidence first suggested that the genome is not a stationary entity but rather is subject to modifications and reorganization, a concept that was met with criticism by the scientific community at the time.

Nowadays, the increasing number of sequenced genomes has revealed that the content of TEs varies among species [2,3]. Overall, vertebrate genomes are largely made up of TEs that contribute to genome size and architecture [4,5,6]. Among them, amphibians and lungfish are the only two taxa having species with the largest genomes due to the considerable impact of mobile elements. Retroelements are mainly present in jawless fish, cartilaginous fish, coelacanths, lungfish, birds, and mammals; on the other side, DNA transposons dominate the genomes of ray-finned fish and anurans. The richness in terms of transposon diversity is another variable characteristic between lineages: teleosts show the highest diversity in terms of TE families, differently from mammals. Moreover, the number of TE copies can be very high in some species and low or even absent in others. For example, L1 and L2 retroelements were lost in birds, while CR1 retroelements are present in many copies in these organisms [7].

TEs have long been considered ‘selfish’ or ‘parasite’ elements because of their ability to replicate themselves. However, it is now recognised that these genetic elements play a key role as drivers of genome diversity and, hence, of speciation, contributing to the evolutionary success of organisms. Indeed, in some cases, TEs can be co-opted or exapted to benefit the host, for example, by creating key adaptations and being a source of genetic diversity and regulatory innovations useful for species evolution [8,9,10]. In many other cases, TE insertions are neutral, and in others, their transcription and transposition might be responsible for the alteration of normal genes leading to negative effects, for example, interrupting coding genes. Diseases like hemophilia, cystic fibrosis, or cancer can be caused by disrupting or enhancing tumor oncogene suppressor genes [11,12]. In addition, TEs derived from exogenous sources (ERV) or from mutated internal mobile elements can invade the host genome. Therefore, TE activity must be regulated by the host genome, which has evolved “defense” mechanisms to suppress their activity. In this review, the main TE silencing mechanisms acting in vertebrates are presented. In particular, we focused on proteins of the Argonaute family (PIWI and AGO) and the small non-coding RNAs accomplishing piwi-interacting RNAs (piRNAs), short interfering RNAs (siRNAs), and microRNAs (miRNAs) [13]. piRNAs are loaded by PIWI proteins to transcriptionally and post-transcriptionally silence transposons in germline cells. siRNAs and miRNAs are associated with AGO proteins and act as piRNA/PIWI systems to prevent the effects of TE transposition in somatic tissues. Another interesting mechanism is represented by the Krüppel-associated box (KRAB) zinc finger (ZNF) proteins (KRAB-ZFPs) [13]. They have zinc fingers at the C-terminus to bind TE sequences and a KRAB domain at the N-terminus to recruit the proteins of the nucleosome remodeling deacetylase complex (NuRD). This system is now known to act at both embryonic and adult stages, not only in sarcopterygians but also in actinopterygians, probably with the involvement of fish-specific key proteins. The Human Silencing Hub (HUSH) complex targets full-length, evolutionarily young, and transcriptionally active L1 retroelements [14]. This system has the ability to recognise intronless RNAs, a feature of retroelements, avoiding the silence of intron-containing genes.

In general, these mechanisms determine an increase in the compactness of chromatin structure through the deposition of epigenetic markers at the DNA and histone levels. In particular, DNA (cytosine-5-)-methyltransferase 1 (DNMT1) and DNA (cytosine-5-)-methyltransferase 3 alpha (DNMT3a) are responsible for transferring methyl groups to CpG residues to establish DNA methylation patterns. At the histone level, proteins involved in heterochromatin formation are SET domain bifurcated histone lysine methyltransferase 1 (SETDB1), the chromobox homolog 5 (CBX5 is also named heterochromatin 1a), the chromobox homolog 1 (CBX 1 is also named heterochromatin 1b), and the chromobox homolog 3 (CBX 3 is also named heterochromatin 1g). SETDB1 trimethylates Lys-9 of histone H3 and this epigenetic mark serves to recruit the CBX proteins. SETDB1 is targeted to histone H3 by TRIM28/KAP1, a factor recruited by KRAB zinc-finger proteins but also by the HUSH complex to favor higher levels of chromatin compactness.

The host genome can fine-tune these mechanisms to allow the coexistence between mobile elements and eukaryotic host-coding genes, avoiding negative effects due to transposition. Moreover, evidence suggests that TEs can be responsible for rewiring tissue-specific regulatory networks and modifying epigenetic patterns. This challenges the general notion that TEs are constitutively silenced; on the contrary, they can be domesticated for the regulation of host-coding gene expression, permitting species adaptation and resilience as well as ensuring human health.

## 2. Classification of Transposable Elements

**Figure 1:**
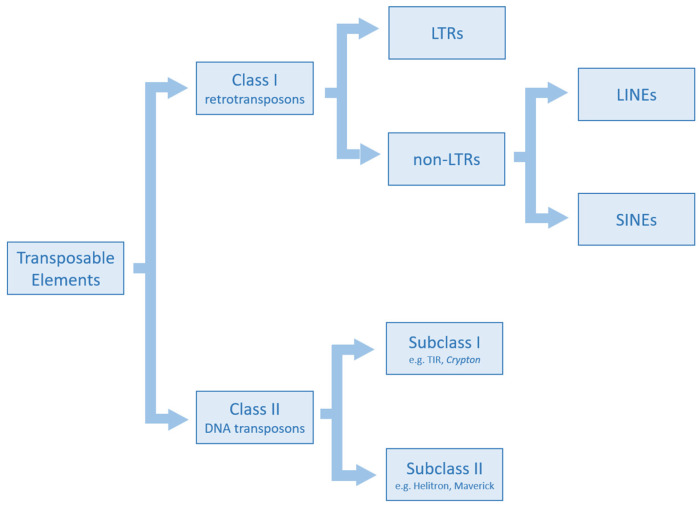
Classification of TEs. The scheme reports the main TE classes and their subgroups.

TEs are repetitive elements interspersed in the genome and they are able to move from one region to another by a transposition mechanism using RNA or DNA molecules as intermediates (Figure 1). In the former case, TEs are called retrotransposons and constitute Class I, while in the latter case, they are named DNA transposons and are part of Class II. Class I elements are characterised by a copy-and-paste transposition mechanism. Their RNA intermediate is reverse transcribed into its complementary DNA by a reverse transcriptase (RT) encoded by the mobile element. After this step, the new copy is integrated into the host genome. Due to their transposition mode, retroelements increase their copy number, and thus they have an impact on genome size. Class I mobile elements are composed of long terminal repeat (LTR) and non-LTR subclasses. LTR retrotransposons present long direct terminal repeats useful for transposition. Their structure, similarly to exogenous retroviruses, includes the *gag* gene that encodes viral structural particles and the *pol* gene that encodes the complete retrotranscription machinery (reverse transcriptase, ribonuclease H, and integrase). In addition to these two genes, exogenous retroviruses possess the *env* gene, which is responsible for the formation of proteins that are part of the viral envelope. However, traces of the *env* gene have also been found in LTR retrotransposons. *Dictyostelium* Intermediate Repeat Sequence (DIRS) are LTR retroelements structurally characterized by a tyrosine recombinase (YR) instead of an integrase and by inverted terminal repeats. Non-LTR retroelements include Long Interspersed Nuclear Elements (LINEs) and Short Interspersed Nuclear Elements (SINEs). The former are autonomous retroelements constituted by two open reading frames (ORFs), of which ORF2 encodes a reverse transcriptase and an endonuclease, and a poly A tail at the 3′ end [15]. SINE retroelements are not autonomous and need RT encoded by autonomous elements to make transposition [16]. Penelope retroelements are part of Class I and present a wide diversity of structures compared to the other elements. The common components are pseudo-LTRs (pLTRs), an RT, and an endonuclease. Class II mobile elements use a DNA intermediate to transpose their copies into a novel chromosomal position. These elements can be divided into subclasses I and II. Subclass I mainly includes TIR and Crypton. TIRs are autonomous elements characterized by terminal inverted repeats (TIRs) and encode a transposase through which they move via a cut-and-paste mechanism in which both DNA strands are cleaved. The DNA transposons hAT, Merlin, Mutator, PiggyBac, PIF-Harbinger, Tc1-Mariner, and Transib are part of this subclass. Crypton elements use a YR in the transposition mechanism, probably involving recombination between a circular intermediate and a DNA target. Helitrons and Mavericks are the two major representative elements of subclass II. They transpose via a copy-and-paste mechanism. In particular, helitrons replicate using a rolling-circle mechanism and encode a replication initiation (Rep) and a DNA helicase (Hel), while Maverick elements encode an integrase, an ORF, and a polymerase B. Miniature Inverted Transposable Elements (MITEs) are non-autonomous elements of Class II, and thus they use transposases encoded by autonomous TEs for transposition [17].

## 3. Regulatory Mechanisms of Transposon Silencing

### 3.1. Argonaute Protein Family and Small Non-Coding RNAs

The main tool used by eukaryotic cells and genomes to counter TE activity is the RNA interference (RNAi) system. The RNAi phenomenon was first discovered in the nematode *Caenorhabditis elegans* [18] and uses short RNA duplexes in which one of the strands is complementary to the mRNA of the target gene to post-transcriptionally silence it. In general, RNAi may act through two modalities: it suppresses transcription by transcriptional gene silencing (TGS) or it activates a sequence-specific RNA degradation process by post-transcriptional gene silencing (PTGS) [19]. The canonical RNAi pathways act at the gene level but also participate in transposon and foreign DNA silencing [20,21,22]. The three key components involved in the RNAi system are the proteins of the Argonaute family (AGO and PIWI), dicer-like proteins, and RNA dependent RNA polymerase (RdRP). By analysing these proteins, it is possible to understand their prokaryotic origin given the presence of archaeal, bacterial, and even viral compositions. The defence function of RNAi against viruses and transposons was present in the last eukaryotic common ancestor [23].

The RNAi system is differentiated between germinal and somatic cells and is mediated by piRNA and siRNA/miRNA, respectively [22]. The structure of eukaryotic Argonaute family proteins is highly conserved and presents four characteristic domains: N-terminal, PIWI-Argonaute-Zwilli (PAZ), Middle (MID), and the RNase H catalytic domain named PIWI. The N-terminal domain participates in the unwinding of the RNA duplex and plays an auxiliary role in targeting RNA cleavage. The PAZ domain is required for binding the 3′ end of the RNA guide, while the MID domain, with its pocket, accommodates the 5′-terminal nucleotide of the RNA guide. The PIWI domain contains the catalytic centre (DEDH/D) and is an endonuclease that allows cutting RNA targets complementary to the small RNA guide [24].

The members of the *Piwi* and *Ago* subfamilies are present in all eukaryotes, while in worms and trypanosomes, lineage-specific duplication events occurred, leading to the worm-specific *WAGO* subfamily and the Trypanosome *AGO* family, respectively [25]. In vertebrates, the *Piwi* subfamily includes *Piwil1* and *Piwil2* in all gnathostomes, *Piwil4* in both cartilaginous fish and sarcopterygians (it is absent in actinopterygians), and *Piwil3* is present only in mammals. Microsyntenic analyses have shown that these genes are located on different chromosomes. In vertebrates, the *Ago* subfamily comprises four members: *Ago1/3/4,* organised in tandem at the same chromosome locus, and *Ago2,* located in a different genomic region. Phylogenetic analyses considering the major evolutionary lineages have revealed that the evolution of the Argonaute family was shaped by whole genome duplications and lineage-specific gains and losses [26].

#### 3.1.1. piRNA and PIWI Proteins: Small RNA Biogenesis and TE Silencing Activity

piRNA/PIWI complexes drive a defence system that silences TEs during germinal development [27,28] through two mechanisms: one in the nucleus, where piRNAs silence TE transcriptional activity by recruiting chromatin remodelers such as DNA methyltransferases (DNMTs) and histone methyltransferases (HMTs); the other in the cytoplasm, where TE-derived piRNAs are recognised and degraded [13]. At the same time, it is important to consider that although germline protection against transposon activity is conserved, piRNA genes have undergone rapid evolution, and their mechanism of biogenesis shows strong phylogenetic diversity. This suggested a co-evolution between TEs and piRNA pathways [29].

piRNAs are the most heterogeneous small noncoding RNAs in animals [30,31,32,33]. They are generated from longer RNA transcripts originating in specific genomic loci, named “clusters” [21,32,34,35,36]. These regions contain a large number and various types of TEs, and thus, the piRNAs generated from them are homologous not only to TEs in the clusters but also to TEs located elsewhere, allowing the repression of TEs in trans. Transcription of piRNA precursor RNAs can occur from a single strand of DNA, uni-strand clusters generating piRNA precursors by canonical unidirectional transcription, or from both strands of DNA, double-strand clusters, identified mainly in some invertebrates as dipterans [21,37,38] and lepidopterans [21,39].

Based on the different mechanisms of 5′ end formation during biogenesis, piRNAs can be classified as primary or secondary piRNAs. In the first mechanism, after transcription, piRNA precursor transcripts move from the nucleus to the cytoplasm by the shuttling protein HMG protein Maelstrom (Mael). Here, they are concentrated in the “nuage,” where they are also protected from nucleases. In addition, this compartmentalisation can also prevent mRNAs and lncRNAs from entering the piRNA pathway. In the nuage, piRNA precursors are processed in close association with mitochondria. In mice, MOV10L1 RNA helicase produces shorter RNAs, and mitoPLD endonuclease transforms precursor piRNAs into intermediate fragments [40]. In the last step, piRNAs are trimmed by PNLDC1/Trimmer localized to the outer membrane of mitochondria [41,42,43,44,45,46,47,48,49], and 2′-O-methylation at the 3′ end is added by mouse Hua Enhancer 1 Homolog 1 (mHen1). The secondary piRNA biogenesis, also known as the “Ping-Pong” amplification loop, is limited by the availability of piRNA precursors [21]. Indeed, this cycle consumes TE transcripts, given that TEs are silenced during the amplification of the primary and secondary piRNA pools [50].

**Figure 2:**
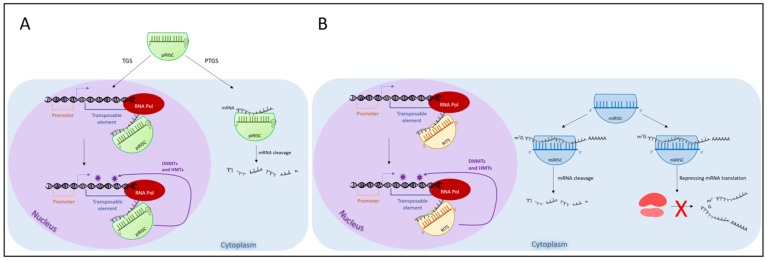
RNA interfering silencing mechanisms. (**A**) action of the piRNA-induced silencing complex (piRISC) at the transcriptional level (TGS) in the nucleus and post-transcriptional level (PTGS) in the cytoplasm. piRISC and piRNAs are coloured in green. (**B**) action of the RNA-induced initiation of transcriptional gene silencing (RITS) complex acting on nascent RNAs in the nucleus and of the miRNA-induced silencing complex (miRISC) that cleaves the mRNA or blocks mRNA translation in the cytoplasm. RITS and endo-siRNAs are coloured orange; miRISC and miRNAs are coloured in blue. Purple stars indicate epigenetic marks deposited by DNMTs and HMTs. For more details, see the main text.

The produced piRNAs are loaded into PIWI proteins to form the mature piRNA-PIWI complex called the piRNA-induced silencing complex (piRISC) [40] (Figure 2A). These complexes use piRNAs to guide PIWI proteins to target RNAs and cleave them or suppress TEs through DNA methylation and histone modifications. In particular, piRNA/PIWI proteins recruit DNMTs and HMTs to TEs. Through the deposition of methyl groups onto DNA cytosines and at specific residues of histone tails, the chromatin structure is compacted, and the target regions are silenced.

In vertebrates, the TE silencing system mediated by piRNA/PIWI complexes has been investigated in several organisms. In teleosts, Houwing and colleagues [51] have shown the activity of *Piwil1* and the presence of piRNAs both in the ovary and testis of zebrafish. Moreover, the involvement of Dicer in the production of these small RNAs suggests that this pathway is widely conserved. In coelacanths, the activity of *Piwi* and that of proteins involved in piRNA biogenesis have been recorded in gonadal and somatic tissues, while in lungfish, it was limited to gonadal tissues [26]. The lower activity of these genes and others involved in TE silencing mechanisms compared to coelacanth suggested that this finding might be in relation to the presence of old TE copies in the giant genome of lungfish. A different condition was recorded in the newt *Cynops orientalis*, in which a higher activity of TEs and silencing mechanisms was detected, probably due to the presence of younger TE copies [52]. In addition, the expression of piRNAs and TEs has been investigated in other vertebrate species, such as those belonging to amphibians, birds, teleosts, reptiles, and mammals, with a wide range of genome sizes evidencing an action in both the ovaries and testes [53,54,55]. Pasquesi and colleagues [56] have tested the activity of TEs and the PIWI pathway not only in gonadal tissues but also in seven somatic tissues of 12 vertebrates. Similarly, Galton and colleagues [57] have reported that in chickens, piRNAs and *Piwil1* are present in the somatic cells of the neural tube and are required for neural crest specification and emigration. Indeed, PIWIL1 targets *ERNI*, a transposon-derived gene that in turn inhibits *Sox2*, which is involved in neural crest specification and epithelial-to-mesenchymal transition. These findings indicate that the piRNAs/PIWI pathway can also be involved in the regulation of somatic development in vertebrates. Vandewege et al. [58] have compared the piRNA/PIWI and TE dynamics in squirrels, organisms characterised by the absence of mobilization of LINE and SINE retroelements, with rabbits and mice. Their data suggested that PIWI proteins act weakly on TEs that do not represent a threat.

#### 3.1.2. miRNA/siRNA and AGO Proteins: Small RNA Biogenesis and TE Silencing Activity

The partners of AGO proteins are miRNA (~22 nt) and endo-siRNA (20–26 nt). The biogenesis of miRNA begins in the nucleus, where they are transcribed by RNA polymerase II as a long transcript called primary miRNA (pri-miRNA) containing hairpin structures with a terminal loop of ~10 nt. The molecules are processed using the microprocessor complex composed of Drosha and DiGeorge Syndrome Critical Region Gene 8 (DGCR8). This latter protein binds the pri-miRNAs through a double-stranded RNA binding domain and facilitates their cleavage by Drosha. The miRNAs precursors obtained move to the cytoplasm thanks to exportin 5 and Ran GTP. Here, the endonuclease Dicer cuts a shorter double-stranded fragment that is incorporated into the miRNA-induced silencing complex (miRISC) that acts as an effector in the miRNA pathway [30] (Figure 2B). On the basis of the complementarity between miRNA and the target, miRISC degrades mRNA or represses mRNA translation [59]. Unlike miRNA, endo-siRNA biogenesis is not strictly related to the nuclear microprocessor, and it is processed starting from long double-stranded RNAs (dsRNAs) [60]. They are produced by Dicer and loaded onto AGO proteins, forming the RNA-induced initiation of transcriptional gene silencing complex (RITS) (Figure 2B). The small RNA is used to target nascent RNAs still attached to RNA polymerase and DNA. These transcripts are cleaved, and heterochromatin in these DNA regions increases through methylation at lysine 9 of histone H3 and at the DNA level.

In vertebrates, the activity of *Ago* genes in relation to TEs has been investigated in teleosts and sarcopterygians in both gonadal and somatic tissues [26,52,56,61,62] as well as in the germline in mammals [63,64]. A role for AGO2 as a genome-defence system against young TEs has been reported in the nucleus of quiescent cells in mice [65]. Piriyapongsa et al. [66] have evidenced that miRNAs derived from TEs are co-opted for the regulatory systems leading to lineage-specific phenotypes contributing to species divergence. In zebrafish, miRNAs target V-SINEs (vertebrate SINEs) inserted in the 3′ UTR of mRNAs and play a role in modulating cell responses to different stimuli. Moreover, the presence of these TEs and related miRNAs in the genome of fish could have favoured the radiation of this taxon [67]. Similarly, in bats, a burst of DNA transposons and derived miRNAs has been proposed as responsible for the diversification of Vespertilionidae [68].

### 3.2. KRAB System

The Krüppel-type C_2_H_2_ zinc finger (ZNF) proteins present an N-terminal KRAB domain and a variable number of zinc finger repeats at the C-terminus. The KRAB domain was originally defined as a “heptad repeat of leucines” [69] and was first described in human ZNF10/Kox1 identified in T cells. Because of its occurrence with the Krüppel-type C_2_H_2_ zinc finger motifs, this domain was then named “KRAB” [70]. The KRAB domain may have evolved from *PRDM9/Meisetz,* which has an ancestral KRAB-like domain at the N-terminus, a central Suvar3–9, Enhancer-of-zeste, Trithorax (SET) domain, which catalyses the trimethylation of lysine 4 in the histone H3, and an array of tandem zinc finger motifs at the C-terminus [71]. This KRAB-like domain is related to the divergent KRAB motif found in the members of the Synovial sarcoma, X breakpoint (SSX) family that do not present zinc fingers [72].

Analyzing more than 200 vertebrate genomes, Imbeault and colleagues [73] have reported KRAB-ZFPs in coelacanths, lungfish, and tetrapods, dating the origin of these sarcopterygian-specific genes to 420 million years ago. In humans, most of the genes encoding KRAB-ZFPs are organised in clusters on 19q, and others are located on both autosomes and sex chromosomes [74].

**Figure 3:**
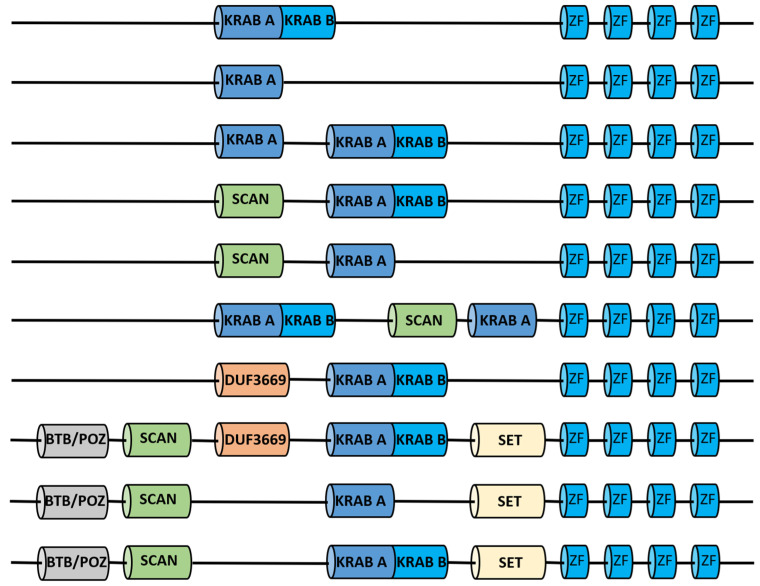
Schematic representation of KRAB-ZFPs and associated domains. For simplicity, only four zinc fingers are shown. KRAB (Krüppel-associated box); ZF (zinc finger); SCAN (SREZBP, CTfin51, AW-1, and Number 18 cDNA); DUF3669 (domain of unknown function 3669); BTB/POZ (Broad-Complex, Tramtrack, and Bric-a-brac/POxvirus and Zinc finger); SET (Suvar3–9, Enhancer-of-zeste, Trithorax).

The KRAB domain, consisting of 75 amino acids, is subdivided into two subdomains that are usually encoded by separate exons (apart from coelacanths, in which it is encoded by a single exon): the KRAB-A subdomain (40–50 amino acids) and the KRAB-B subdomain (20–25 amino acids), which also exists in various forms called B, b, BL, and C [75]. A small subset of members of this family contains other domains such as the SCAN domain, responsible for oligomerization with other SCAN-containing proteins [74,76,77], Broad-Complex, Tramtrack, and Bric-a-brac/POxvirus and Zinc Finger (BTB/POZ) domains acting as dimerization motif, the SET domain, and DUF3669, whose function is still unknown [78] (Figure 3).

**Figure 4:**
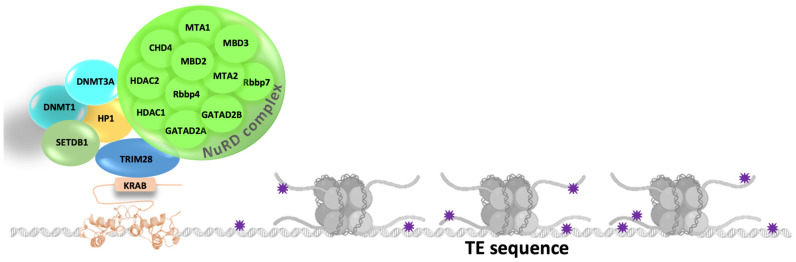
TE silencing mechanism based on KRAB-ZFPs. Purple stars represent the methylation of DNA sequences and histone tails.

KRAB-A is involved in the repressive activity that is enhanced by the KRAB-B domain (Figure 4). The KRAB-A interacts with Tripartite Motif protein 28 (TRIM28, also named KRAB-Associated Protein 1 (KAP1) or Transcription Intermediary Factor 1-Beta (TIF1b)), which in turn acts as a scaffold for recruiting proteins involved in heterochromatin formation such as histone H3K9 methyltransferase SETDB1, heterochromatin protein HP1, and the NuRD complex. TRIM28 dimerizes and binds a single KRAB-ZFP through the RING, B-box zinc finger, and Coiled-Coil (RBCC) domains located at the N-terminus. Some residues have been identified as crucial for KRAB binding and transcriptional silencing activity [79,80]. The PxVxL motif of TRIM28 recruits HP1 and the DNA Methyltransferases (DNMT1 and DNMT3A). In the C-terminal region, TRIM28 presents a PHD bromodomain that binds the SUMO E2 ligase Ubc9 to post-translationally SUMOylate lysine residues in the bromodomain to recruit and activate the histone methyltransferase SETDB1 and the NuRD complex [79]. The former adds the H3K9me3 mark, while the latter is a chromatin remodeling complex that contains the histone deacetylases 1 and 2 (HDAC1 and HDAC2), the chromatin helicase DNA binding protein 4 (CHD4), the retinoblastoma-binding proteins 4 and 7 (Rbbp4 and Rbbp7), the zinc-finger proteins GATA Zinc Finger Domain Containing 2A or 2B (GATAD2a and GATAD2b), two Metastasis-associated Proteins (MTA1, MTA2, and/or MTA3), and the Methyl-CpG Binding Domain Protein 2 or 3 (MBD2 or MBD3).

The zinc finger motifs of KRAB-ZFPs are involved in binding DNA sequences by interacting with three nucleotides of the primary DNA strand (via amino acids at positions 1, 3, and 6 of the C2H2 helix) and some nucleotides on the secondary strand (via amino acid 2). The amino acids contacting DNA are under positive selection to evolve rapidly to efficiently target mutable DNA sequences such as TEs or viruses [81].

Indeed, KRAB-ZFPs have been reported to be implicated in several biological events such as embryonic development, cell differentiation, cell proliferation, cell cycle regulation, and TE silencing [82,83]. They act mainly on retrotransposons and endogenous retroviruses in early embryonic development [73,84,85]. Indeed, during this developmental window, methylation is reduced and the chromatin state is reprogrammed to allow the acquisition of totipotency by the zygote [86]. The low level of methylation and chromatin compactness may allow TE transposition, with consequent effects due to new insertions. Recent papers have reported that the KRAB-related TE silencing mechanism seems to be active in adult tissues as well. Ecco and colleagues [87] have demonstrated that this system acts in murine differentiated tissues, with effects also on the expression of genes located close to TEs. This suggested that KRAB-ZFPs and TEs might be involved in regulating many physiological events, and thus the KRAB system is not only a silencing mechanism but also a tool for TE domestication for the benefit of the host [88,89]. This has also been proposed by Grassi et al. [90] to fine-tuning regulate the expression of coding genes in the brain. Indeed, in primates, the presence of lineage-specific TEs might have contributed to the complexity of this organ.

The transcriptional activity of TEs and genes involved in NuRD complex composition and recruitment was also detected in the gonads of adult specimens of two basal sarcopterygians (coelacanths and lungfish) and the urodele *C. orientalis* (having a giant genome as dipnoans) [52]. Wang and colleagues [53] have investigated the relationship between TE activity and silencing mechanisms in 15 vertebrate species with giant genomes. Their data suggested that differences in the expression of genes involved in these systems are related to the evolutionary phase in which the genome is: TE silencing is active during genome contraction while it is reduced during genome expansion. The patterns of expression of both TEs and genes involved in NuRD complex composition and recruitment were also investigated in somatic and gonadal tissues of 12 vertebrate species belonging to the major lineages, revealing variations across species and tissues [56].

KRAB-ZFPs and TEs coevolve as a result of the “arms race” model, according to which TEs change by acquiring mutations to escape the repression made by KRAB-ZFPs. Consequently, some of these KRAB-ZFPs evolve rapidly, modifying their zinc fingers to be able to recognise the escaped TEs [91]. This should have determined the expansion in the number of KRAB-ZFPs in higher vertebrates [92]. In humans, KRAB-ZPFs represent one-third of about 800 different zinc finger proteins and are one of the largest families of transcriptional regulators [93].

Although zinc finger proteins encoding the KRAB domain and TRIM28 are absent in fish, Carotti and colleagues [61,62] proposed the presence of a zinc finger protein showing a KRAB-like domain in teleosts and suggested TRIM33 as a functional substitute for TRIM28. Indeed, in actinopterygians, KRAB-like proteins in concert with TRIM33 may recruit the NuRD complex and other proteins involved in heterochromatin formation, functioning as KRAB-related TE silencing mechanisms in sarcopterygians. The activity of TEs and that of this system in organisms whose physiology is strongly influenced by abiotic factors such as temperature and salinity suggest that a cross-talk between TEs and the KRAB system might exist and might represent a regulatory tool used by the genome to face environmental changes and allow organism adaptation.

### 3.3. Human Silencing Hub Complex

**Figure 5:**
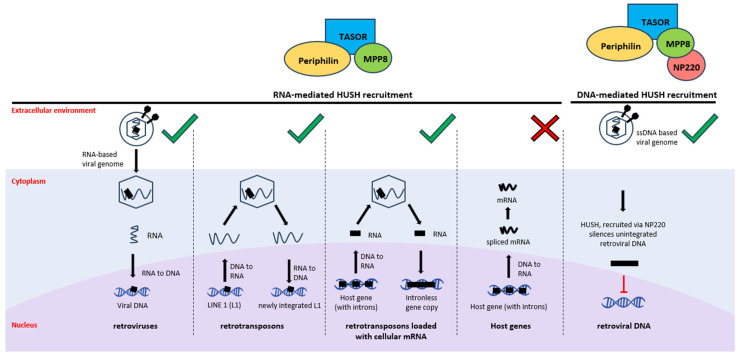
RNA- and DNA-mediated Human Silencing Hub (HUSH) recruitment. On the left, RNA-mediated HUSH recruitment is reported. The HUSH complex, composed of transcription activation suppressor (TASOR), Periphilin, and M-phase phosphoprotein 8 (MPP8), is able to silence retroviruses, retrotransposons, and retrotransposons carrying cellular mRNA (as indicated by green thick). Host genes, characterised by the presence of introns, are able to overcome HUSH-mediated silencing (as indicated by the red cross). On the right, DNA-mediated HUSH recruitment via DNA-binding nuclear protein 220 (NP220) is shown. This system acts on retroviral DNA, preventing its integration into the host genome.

Despite the fact that invading pathogens represent a constant threat to eukaryotic genomes, DNA derived from the integration of genetic parasites can also be beneficial for the host genome. Indeed, it represents raw genetic material that can be co-opted and contribute to the host genome’s evolution through domestication [94]. As recently reviewed by Seczynska and Lenher [95], how the immune system can distinguish between foreign and self-nucleic acids remains unclear. The discovery of the Human Silencing Hub (HUSH) complex started to answer open questions concerning how the host genome can discriminate between retroelement-derived DNA from its own genome and control its activity (Figure 5). The HUSH complex was first discovered in the Lehner laboratory while performing studies on position-effect variegation, i.e., how an identical gene can be differently expressed as a result of its position due to epigenetic silencing [96]. This phenomenon can be found from fish to mammals [97].

The HUSH complex is a stable system composed of three main proteins: transcription activation suppressor (TASOR), M-phase phosphoprotein 8 (MPP8), and Periphilin. In addition, two effectors are present: MORC family CW-type zinc finger 2 (MORC2), an ATP-dependent chromatin remodeler that increases its compactness, and SETDB1, which acts in the deposition of histone 3 lysine 9 trimethylation at the target loci [97]. The recruitment of the HUSH complex starts with the binding of Periphilin to the nascent RNA. MPP8 then recruits SETDB1 thanks to an interaction with the nuclear chaperone Activating Transcription Factor 7 Interacting Protein (ATF7IP) [95]. A delicate equilibrium regulates the reading activity of MPP8 and the writing activity of SETDB1. Recognizing preexistent trimethylations, MPP8 recruits ATF7IP-SETDB1 at the target locus to allow the deposition of further epigenetic markers by SETDB1 [97,98,99].

However, MPP8 is not essential for repression. MPP8 contributes to maintaining in a stable manner the HUSH complex at the locus [14] as well as the binding of Periphilin to the target RNA. Mimicking long interspersed nuclear element 1 (LINE1, L1) invasion with reporter transgenes, the Lehner laboratory discovered that the key role of the HUSH complex is to target specifically full-length, evolutionarily young, and transcriptionally active L1s. Moreover, this complex avoids the repression of host genes thanks to its ability to recognise the presence of introns [14]. Indeed, the lack of introns is an intrinsic feature of retroelements. What enables HUSH to distinguish intronless invading elements from intron-containing genes is the specific binding between Periphilin and nascent RNA. “Specific” refers to the selective binding of HUSH to L1 transcripts, KRAB-ZF genes, and HUSH-sensitive loci [14]. Only active, full-length, and evolutionarily recent L1 can be silenced using the HUSH complex [100] and represent RNA-mediated HUSH silencing (Figure 5).

Beside this canonical silencing method, DNA-mediated HUSH recruitment can also be found, in which the nuclear protein 220 (NP220) acts as an intermediate. NP220 is a dsDNA-binding protein and is characterised by a DNA-binding domain and a single C-terminal ZNF motif. Zhu and colleagues [101] have evidenced that MLV DNA was bound by NP220, marked with histone deacetylation, and thus silenced by the HUSH complex.

HUSH is not only involved in the control of the reverse flow of genetic information [95]. Recently, cooperation between HUSH and Nuclear Exosome Targeting (NEXT) complexes has been reported by Garland and colleagues [102]. The NEXT complex components are the zinc finger CCHC-type containing 8 (ZCCHC8) and the RNA-binding RBM7 proteins. The NEXT complex cooperates with HUSH to restrict transposon expression. Elevated TE levels resulted from the knockout of ZCCHC8 in mouse embryonic stem cells. The authors highlighted an interaction between ZCCHC8 of NEXT and the MPP8 protein that is fundamental for the recruitment of NEXT by the HUSH complex to chromatin at MPP8-bound TE loci [102].

## 4. TE Silencing Mechanisms as Controlling Tools for Vertebrate Adaptation

TEs differ in total content as well as in TE typology and family diversity between vertebrate lineages [6,103]. They strongly contributed to the evolution of this taxon, creating crucial innovations that allowed vertebrate diversification and adaptation [8,9,10,104]. Indeed, TE transposition might create raw genetic material on which natural selection can act, contributing to the evolution of species-specific traits. However, in many cases, TEs cause genome instability, and consequently, protective mechanisms were evolved to prevent TE transposition and allow the coexistence of TEs and eukaryotic host-coding genes. On the other side, an increasing number of papers attribute to these mobile elements a role as a source of genetic and epigenetic variability with adaptive potential. This implies that TEs are not elements merely to silence but to include in the regulatory pathways of the host. The co-option of TEs as *cis*-regulatory elements, such as promoters or enhancers, of host genes is becoming increasingly apparent [105,106,107]. Indeed, TEs may contain sequences involved in regulating themselves but also nearby genes in a tissue-specific fashion [8,108,109,110]. Xie and colleagues [111] have shown that TEs are not constitutively silenced, but their repression differs between human cell types. Moreover, their data suggested that hypomethylated TEs gain enhancer signatures, indicating that these elements can mediate gene regulation in relation to epigenetic silencing systems. Several TE-derived promoters and enhancers have been reported in embryonic stem cells of mice [112], as well as in rodent placenta [8], in the innate immune system in humans [105], and in the mammalian brain [90]. Moreover, old TEs are more likely to present transcription factor binding sites or CTCF-binding sites to reorganize the 3D chromatin structure since they escape DNA methylation silencing due to CpG deamination [113]. TEs can regulate the gene expression of host-coding genes, considering their position and the mechanisms involved in their silencing. Chen and colleagues [114] have demonstrated that the KRAB system also has an activation function for neighboring genes involved in adipogenesis by targeting a retroelement located upstream that functions as an enhancer. Therefore, the coevolution between KRAB-ZFPs and TEs is not only the result of the arms race model but probably also the domestication of TE-derived regulatory sequences, which has had an important impact [89,110]. In somatic tissues, this ability of TEs to rewire gene expression networks could represent a tool to cope with environmental changes, allowing species adaptation. Organisms are continuously threatened by changes in environmental factors, with a consequent increase in selection pressure and evolutionary rate. In the context of species adaptation, this leads to the onset of new phenotypes that are well-adapted to the new conditions and able to improve the fitness of organisms. It is known that TEs are environmentally sensitive molecular elements whose activity can be triggered by numerous environmental stressors [115]. Recently, in vertebrates, several papers have reported variations in TE transcriptional levels in response to changes in biotic and abiotic factors accompanied by the activation of genes involved in TE silencing mechanisms [61,62]. This relationship suggests that controlling systems and TEs cooperate to create an epigenetic regulatory pathway that modulates physiological events in response to environmental changes to allow species adaptation. Therefore, the genome might be able to balance the action of these mechanisms to exploit the TE activity for host advantage and not merely silence it.

Understanding the biological functions of TEs is also important for human health, as variations in the activity of these elements and of their controlling mechanisms have been reported in association with disease and cancer [116,117,118].

## 5. Conclusions

TEs have contributed to vertebrate diversification by creating innovations, for example, in immune system, placental formation, and brain development. Moreover, these mobile elements are one of the most intriguing components of the genome, given their potential role in the genome responsiveness to external stimuli. As here reviewed, TEs and related silencing systems can mediate gene regulation by modulating physiological functions that allow organisms to cope with environmental variations. Indeed, sequences derived from TEs can be regulatory elements (such as enhancers and promoters) that rewire gene expression networks in a tissue-specific manner. This view suggests that TEs are not constitutively silenced, but are regulated by the host genome. In line with these new insights, research efforts are focusing on the evaluation of the TE impact as a key molecular tool for adaptation and resilience. In the framework of climate change, results in this field acquire greater importance, considering endangered species or organisms more susceptible to environmental changes. Moreover, to get more information on this topic as well as uncover additional TE regulation modes, high-throughput sequencing techniques, and bioinformatic pipelines have to improve to produce long reads that contain large repetitive regions together with their flanking regions, useful for better TE annotation [119].

## Data Availability

Not applicable.
